# Osteogenesis effects of magnetic nanoparticles modified-porous scaffolds for the reconstruction of bone defect after bone tumor resection

**DOI:** 10.1093/rb/rbz019

**Published:** 2019-05-29

**Authors:** Ming Li, Jianheng Liu, Xiang Cui, Guofei Sun, Jianwei Hu, Sijia Xu, Fei Yang, Licheng Zhang, Xiumei Wang, Peifu Tang

**Affiliations:** 1 Department of Orthopaedics, Chinese PLA General Hospital, Beijing 100853, China; 2 Beijing National Laboratory for Molecular Sciences, State Key Laboratory of Polymer Physics and Chemistry, Institute of Chemistry, Chinese Academy of Sciences, Beijing 100190, China; 3 State Key Laboratory of New Ceramics and Fine Processing, School of Materials Science and Engineering, Tsinghua University, Beijing 100084, China

**Keywords:** bone tumor, magnetic nanomaterials, PLGA scaffold, bone repair

## Abstract

The treatment of bone defect after bone tumor resection is a great challenge for orthopedic surgeons. It should consider that not only to inhibit tumor growth and recurrence, but also to repair the defect and preserve the limb function. Hence, it is necessary to find an ideal functional biomaterial that can repair bone defects and inactivate tumor. Magnetic nanoparticles (MNPs) have its unique advantages to achieve targeted hyperthermia to avoid damage to surrounding normal tissues and promote osteoblastic activity and bone formation. Based on the previous stage, we successfully prepared hydroxyapatite (HAP) composite poly(lactic-co-glycolic acid) (PLGA) scaffolds and verified its good osteogenic properties, in this study, we produced an HAP composite PLGA scaffolds modified with MNPs. The composite scaffold showed appropriate porosity and mechanical characteristics, while MNPs possessed excellent magnetic and thermal properties. The cytological assay indicated that the MNPs have antitumor ability and the composite scaffold possessed good biocompatibility. *In vivo* bone defect repair experiment revealed that the composite scaffold had good osteogenic capacity. Hence, we could demonstrate that the composite scaffolds have a good effect in bone repair, which could provide a potential approach for repairing bone defect after bone tumor excision.

## Introduction

With the continuous development of imaging modalities, surgical technology, radiotherapy and chemotherapy, 90% patients with long bone tumors have salvage limbs [1]. However, subsequently the resulting problem of bone defects reconstruction still remain a huge challenge. At present, a wide range of treatment options have been reported for osseous defect after the excision of malignant bone tumors, including autografts, allografts, vascularized fibular grafts, artificial prosthesis and devitalized tumor-bearing [2]. Autografts, as the ‘gold standard’ for bone transplantation, have good osteoconductivity and osteoinductivity while avoiding the immunoreaction and the risk of disease transmission [3]. Allografts come from a wide range of sources with good bone conduction [4]. Vascularized fibular retain the blood supply and accelerate the healing of bone defects [5]. Artificial prosthesis could retain the integrity and function of the limb with good mechanical strength [6]. Devitalized tumor-bearing bones preserved osteoconductivity and provide as a less expensive alternative [7]. Although each method has its unique advantages in repairing bone defects, there is a drawback in all of them under the condition of tumor resection, which has no ability to inhibit tumor recurrence. Radiotherapy and chemotherapy can lead to apoptosis of normal cells and cause serious side effects. Therefore, effective inhibition of tumor growth while promoting bone defect repair is the key to the treatment of bone defect after bone tumor resection.

As the fifth largest tumor treatment method approved by Food and Drug Administration (FDA) [8], hyperthermia can effectively prevent tumor metastasis and reduce the chance of recurrence. Magnetic hyperthermia has attracted extensive attention because of its unique advantages in converting electromagnetic energy into heat energy to achieve targeted hyperthermia and heating tumors from the inside out to avoid damage to surrounding normal tissues [9]. Currently, magnetic hyperthermia is mainly based on the role of magnetic nanoparticles (MNPs), which are mostly Fe_3_O_4_ particles and the diameter is between 10 and 20 nm [10]. MNPs can generate higher heat under a smaller magnetic field that means it is conducive to improving tumor thermal efficacy [11]. It has been confirmed that local injection of MNPs has the antitumor ability without affecting the surrounding tissues [12–14], but rare research has been studied the application of bone defect reconstruction with MNPs.

Poly(lactic-co-glycolic acid) (PLGA) is a biocompatible copolymer approved by FDA. Owe to superior mechanical properties, adjustable degradation time and nontoxicity, it has been widely used in bone tissue engineering [15]. Hydroxyapatite (HAP), as one of the major components of native bone, has been widely used to bone tissue engineering because of favorable osteoconductivity [16]. Numerous studies have manufactured a composite scaffold fabricated with HAP, growth factors or drugs conjugated PLGA and confirmed it can effectively promotes bone formation and improve fracture healing [17–19]. Recent studies have showed that incorporation of MNPs within bioceramic materials and polymer had significant effects on cell adhesion, proliferation and differentiation. In particular, addition of MNPs could promote osteoblastic activity and bone formation [20–22]. Therefore, combine MNPs with bone repair scaffolds is considered to improve the performance of bone repair and to extend the applications of hyperthermia for bone defect after bone tumor resection.

Based on the HAP composite PLGA scaffolds we prepared earlier [23], which possessed strong mechanical property and good biocompatibility, endowing it with better performance of bone repair and regeneration at an early stage. Here, we produced an MNPs and combine it with the scaffold described above. Then we preliminary examined the magnetic and thermal properties of the magnetic nanomaterials and emphatically verified the biocompatibility and bone repair ability of the composite scaffolds.

## Materials and methods

### Materials

L-Lactide and glycolide were purchased from J&K Scientific Ltd Iodine, followed by further purification via recrystallization from ethyl acetate twice. Sn(Oct)_2_ was bought from Sigma (Germany). Ferric acetylacetonate, tricalcium phosphate powders, calcium acetate hydrate and trimethyl phosphate hydrate were purchased from the Sigma-Aldrich. NaCl particles, ethanol, fetal bovine serum, ethylene diamine tetraacetic acid (EDTA) and CCK-8 were purchased from J&K Scientific Ltd Iodine. Mouse embryos osteoblast precursor cells (MC3T3-E1) and human osteosarcoma cells (MG-63) were bought from the academy of military medical sciences. New Zealand rabbit, ketamine and penicillin were provided by Chinese PLA General Hospital. Other compounds were purchased from Beijing Chemical Reagents Company, China. All reagents were employed as received unless otherwise noted.

### Preparation of MNPs modified-porous scaffolds

#### Preparation of carboxylated PLGA

PLGA was prepared via ring-opening polymerization of L-lactide and glycolide (nLA:nGA = 50:50). Typically, rigorously dried hexadecanol (0.14 mmol), stannous octoate (0.05 wt% of lactide and glycolide), lactide (50 mmol) and glycolide (50 mmol) were transferred to a glass tube. After purging with nitrogen three times, the tube was sealed under vacuum and heated at 180°C for 20 h. The obtained product was purified by dissolving in chloroform and reprecipitating from ethanol, followed by drying under vacuum at room temperature to achieve pure PLGA. Then PLGA was dissolved in chloromethane and succinic anhydride in the presence of pyridine, the solution was continuously stirred for 4 h. Vacuum distillation removed the dichloromethane and precipitated in methanol for three times, followed by drying under vacuum at room temperature to achieve terminal carboxylated PLGA (PLGA-COOH).

#### Preparation of nano-HAP particles (nHAP)

1.365 g calcium acetate hydrate and 4 g cetyl trimethylamine bromide (CTAB) were dissolved in 125 ml water at 70°C to obtain solution, the same way as 0.985 g tripotassium phosphate monohydrate and 4 g CTAB. Both solutions were filtered by 220 nm water-soluble membrane and then mixed stirring for 15 s before centrifuge. The precipitate was put into 62.5 ml ammonia citrate solution with a pH of 9–10 and stirred overnight. Wash with hot water for five times after centrifugation and freeze dry for 24 h. Calcinate at 800°C for 20 min, followed by washing with distilled water twice at room temperature to achieve nano-HAP particles.

#### Preparation of superparamagnetic nanometer particles

The magnetite (Fe_3_O_4_) nanoparticles used here were prepared by coprecipitation method as previously described [24]. Ferric acetylacetonate was dissolved in benzyl ether in the presence of oleic acid, oleylamine and hexadecanediol. The solution was heated to 200°C for 2 h under nitrogen environment, when the solution from orange brown gradually became dark brown, and then raise the temperature to 300°C for 1 h. Then natural cooling to room temperature, pour it into 40 ml ethanol. A black precipitate of magnetite (Fe_3_O_4_) was produced. Precipitate was extracted and added into a bit of *n*-hexane, following add 0.02 ml oleic acid and oleamine once more to remove impurities by centrifugation, washed three times and dispersed in *n*-hexane.

#### Preparation of PLGA-COOH/HAP scaffold (PLGA/nHAP)

PLGA-COOH (2 g) was dissolved in 50 ml of dioxane, followed by addition of HAP (5 g). After stirring vigorously for 24 h to form a uniform slurry, the preserved NaCl particles (150–300 mm) (100 g) were added with agitation. The homogeneous slurry was then casted into polytetrafluoroethylene molds. The casted slurry was lyophilized after freezing in liquid nitrogen to remove any remaining solvent. The resultant products were then immersed in distilled water for 24 h to dissolve the salt. The distilled water was renewed every 3 h until no chloric ion could be detected via dropping of AgNO_3_ aqueous solution. The fabricated HAP/PLGA scaffolds were dried and kept in a desiccator for further use.

#### Preparation of PLGA-COOH/HAP scaffold loaded with Fe_3_O_4_ (PLGA/Magnetic Fe_3_O_4 _(MF)-nHAP)

PLGA-COOH (2 g) was dissolved in 50 ml of dioxane, followed by addition of HAP (5 g). After stirring vigorously for 24 h to form a uniform slurry. About 10 ml of *n*-hexane dispersion liquid of Fe_3_O_4_ was added, ultrasound 10 min to spin off the solvent. The preserved NaCl particles (150–300 mm) (100 g) were added with agitation. The homogeneous slurry was then casted into polytetrafluoroethylene molds. The casted slurry was lyophilized after freezing in liquid nitrogen to remove any remaining solvent. The resultant products were then immersed in distilled water for 24 h to dissolve the salt. The distilled water was renewed every 3 h until no chloric ion could be detected via dropping of AgNO_3_ aqueous solution. The fabricated HAP/PLGA scaffolds loaded with Fe_3_O_4_ were dried and kept in a desiccator for further use.

### Characterization and mechanical properties of MNPs modified-porous scaffolds

#### Magnetic and thermal properties test

PLGA/MF-nHAP lamina was prepared for vibrating sample magnetometer (EZ7, MicroSense) to test the magnetic saturation intensity. Magnetic thermal effect was analysed by magnetic heatmeter (DM100, nB nanoScale Biomagnetics). The prepared superparamagnetic trioxide nanoparticles were placed in an alternating magnetic field with a frequency of 500 kHz and measured the temperature at 5 mT.

#### Nuclear magnetic resonance spectroscopy detection

The PLGA/MF-nHAP scaffold was detected by nuclear magnetic resonance (NMR) (Bruker Avance 400 III). The phase imaging characteristics of nuclear magnetic T2 were observed.

#### Detection of scaffold microstructure

Scanning electron microscope (SEM) (JSM-6700F, Shimadzu Corporation of Japan) and transmission electron microscope (TEM) (JEM-2100F, Electronics Co., LTD, Japan) test was used to observe the morphology, particle size and measure the pore size of scaffolds. The scaffolds were scanned with Micro-CT Inveon MM CT (SIEMENS, Munich, Germany) with an X-ray voltage of 80 kVP and an anode current of 500 μA. A resolution of 13.5 μm was obtained with 360 steps over 360° rotation. The scanning image of the material was reconstructed in 3D with Micro-CT software. PLGA/MF-nHAP made into thin film to detect the distribution of element with energy dispersive X-ray spectroscopy (EDX) (JSM-6700F, Shimadzu Corporation of Japan).

#### Determination of porosity of scaffolds

The porosity of the scaffolds was measured through the method that we reported previously. Scaffolds was cutted into a cube shape, measured the size of the sample including the height (*H*), length (*L*) and width (*W*) and the weight (*M*_s_). The pycnometer, which was filled with ethanol with density *ρ*_e_ at 30°C, was weighed as *M*_1,_ before a sample of the scaffold with weight *M*_s_ was immersed into the pycnometer and weighed as *M*_2_. Then, the parameters of the samples including the porosity (*ε*), the volume of the scaffold skeleton (*V*_S_) and the volume of whole scaffolds (*V*_w_) were calculated:

$$\begin{array}{l} V_{\text{w}}=H\times L\times W\\ V_{\text{S}}=\;\left(M_{\text{1}}-M_{\text{2}}+M_{\text{S}}\right)/\rho_{\text{e}}\\ \varepsilon =\text{1}-V_{\text{s}}/V_{\text{w}} \end{array}$$

#### Detection the mechanical property of scaffold

The compressive strength measurements were performed on cylindrical scaffolds *in vitro* (diameter =15 mm, height =20 mm). An electronic universal tester (Instron 3366, Instron Corporation) was used to complete the mechanical measurements at a crosshead speed of 5 mm/min at room temperature. Five replicates were tested for each measurement (*n* = 5).

### Evaluation of MNPs modified-porous scaffolds by cell assay *in vitro*

#### *In vitro* tumor cell thermotherapy

MG-63 cells were used to evaluate *in vitro* antitumor ability of magnetite nanoparticles. MG-63 cells (1 × 10^5^) were co-culture with nanoparticles (100 μg/ml) in 96-well plates for 24 h, and then those were placed in an alternating magnetic field with a frequency of 500 kHz for 30 min. The MG-63 cells were washed three times with phosphate-buffered saline (PBS), detached with 2.5% trypsin-EDTA solution and collected by centrifugation (2000 rpm × 5 min), then fixed with 1.5% glutaraldehyde in PBS at 4°C for 30 min. The fixed cells were washed three times with PBS and then 1% osmium tetroxide in PBS was added for 1 h at room temperature. After another three washing steps in PBS, the cells were dehydrated with ethanol three times. Thereafter, the cells were infiltrated with Epon resin and embedded in 100% resin at 60°C for 2 days. Ultrathin sections (70-nm thick) were cut on an Ultramicrotome, stained with lead citrate and observed by TEM. Meanwhile, CCK-8 assay was used to detect the viability of cells, the CCK-8 solution (10 μL) was added to each well, the MG-63 cells were incubated under 5% CO_2_ at 37°C for 3 h. The absorbance of the culture medium was obtained at 570 nm using a micro-plate reader.

#### *In vitro* cell adhesion and proliferation

MC3T3-E1 cells were used to evaluate *in vitro* cell adhesion and proliferation on scaffolds without magnetic field. Scaffolds of specific sizes (diameter = 5 mm, height = 2 mm) were put into wells of a 96-well culture plate after being sterilized with ^60^Co radiation (16 kcy) for 90 min. Then, 100 ml of cell suspension that contained a-minimum essential medium (MEM) supplemented with 10% fetal bovine serum and a cell density of 1 × 10^5^/ml was seeded onto each scaffold. After the cell-seeded scaffolds were maintained under 5% CO_2_ at 37°C for 6 h, changing the medium every 48 h. The attachment of MC3T3-E1 cells cultured on scaffolds for 6 days was captured by SEM. The proliferation of MC3T3-E1 cells cultured on scaffolds for 1, 3 and 7 days was measured using the CCK-8 assay. At each predetermined interval, the original culture medium in each well was removed, followed by addition of the same amount of fresh culture medium. After the CCK-8 solution (10 μL) was added to each well, the MC3T3-E1 cells were incubated under 5% CO_2_ at 37°C for 3 h. The absorbance of the culture medium was obtained at 570 nm using a micro-plate reader.

### Evaluation of MNPs modified-porous scaffolds by rabbit’s ulna defect *in vivo*

#### Surgical procedures

Twenty-four young New Zealand rabbits (provided by the animal center of Chinese PLA General Hospital; female; 2.1–2.6 kg body weight) were randomly divided into three groups (G1: blank, G2: PLGA/nHAP, G3: PLGA/MF-nHAP). Before surgery, the rabbits were anesthetized by intramuscular injection of ketamine (10 mg kg^−1^). After the region of surgery was unhaired and disinfected by iodine, the length of 15 mm bone defect was created in rabbit’s right ulna using oscillating saw with continuous water flow to prevent injury of surrounding soft tissue. PLGA/nHAP and PLGA/MF-nHAP scaffolds were implanted into bone defect sites of each rabbit respectively, while nothing was implanted was blank. After implantation, the muscle and skin were sealed and intramuscularly injected with penicillin (400 000 units every day) lasted one week after surgery. Rabbits were executed and anatomized at 3 months later. The specimens of ulna were stored at −80°C for further characterization. All experimental animal procedures conducted in the present study were approved by the Animal Care and Use Committee of the General Hospital of Chinese People’s Liberation Army.

#### X-ray examination of repaired bone defect

All experimental animal were anesthetized by intramuscular injection of ketamine (10 mg kg^−1^) and scanned using x-ray examination (120 kV, 80 mA, 0.12 s) at every desired interval (4, 8 and 12 weeks) to evaluate mineralization and osteogenesis.

#### Micro-CT scan of repaired bone defect

The specimens of ulna repaired for 12 weeks were scanned using a Micro-CT instrument (Inveon MM CT, Siemens) and performed 3 D reconstruction. The parameters of bone volume/total volume (BV/TV) of the ulnas was analysed by the software attached to the Micro-CT instrument.

#### Histology analysis

After Micro-CT imaging, the specimens were decalcified in 12.5% EDTA, dehydrated by a graded series of alcohol and embedded in paraffin. Moreover, 4 mm thick sections were then obtained and stained with haematoxylin and eosin (H&E) and masson. The resulting histological sections were observed using an optical microscope.

### Statistical analysis

Data were presented as means ± standard deviation for continuous data or as frequencies for categorical data that analysed with SPSS 20.0 (SPSS, IL, USA) software. Differences between groups were analysed using the standard Student’s t-test. P-Values less than 0.05 were considered statistically significant.

## Results

### Characterization of composite scaffolds

#### Morphology of magnetic particles and scaffolds

**Figure 1:**
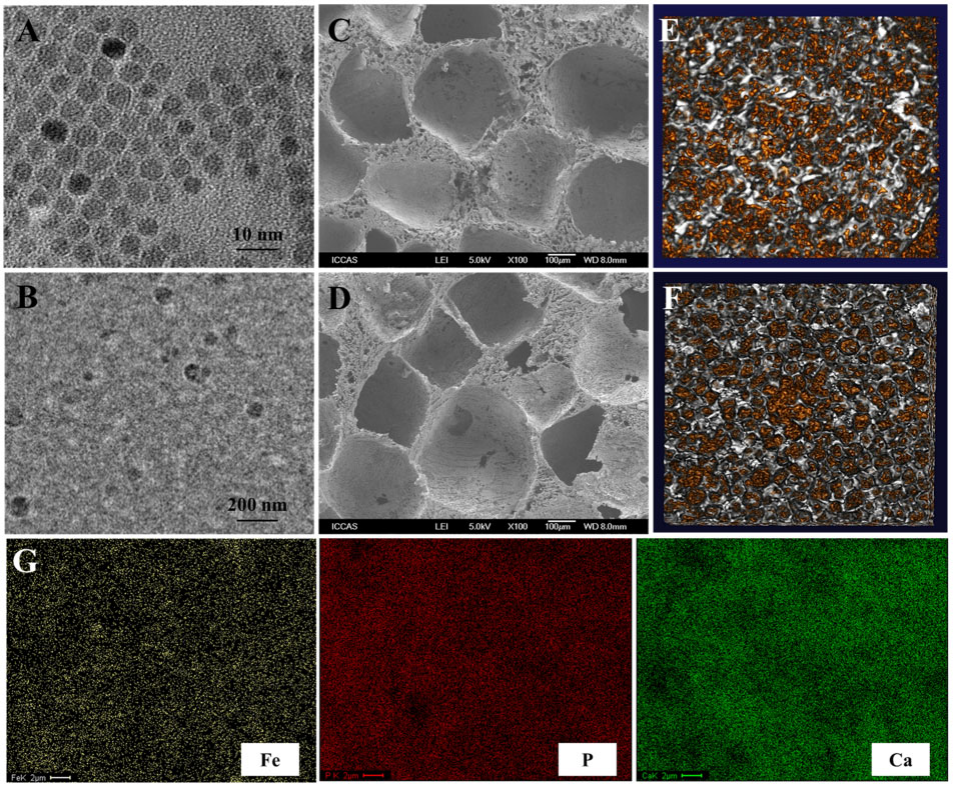
Morphology of scaffolds. TEM images of (A) Fe_3_O_4_ particle and (B) HAP particle. SEM images of (C) PLGA/nHAP and (D) PLGA/MF-nHAP scaffold. 3D reconstruction image of (E) PLGA/nHAP and (F) PLGA/MF-nHAP scaffold. Characterization of element homogeneity by EDX (G).

TEM images of the superparamagnetic Fe_3_O_4_ particle size was 5 nm with good homogeneity (Fig. 1A), the size of HAP particle was 50 nm (Fig. 1B). SEM images of the PLGA/nHAP and PLGA/MF-nHAP showed that they possessed uniform perforative porous structures comprising 150–200 μm macropores (Fig. 1C and D). Micro-CT showed PLGA/nHAP and PLGA/MF-nHAP have porous structure and their porosities were greater than 85% (Fig. 1E and F). Quantitative analysis of porosities was 89.9 ± 1.9% and 89.7 ± 2.0%, respectively. Element homogeneity characterization showed that the distribution of Fe, P and C was relatively uniform (Fig. 1G).

#### Magnetic and thermal properties

**Figure 2:**
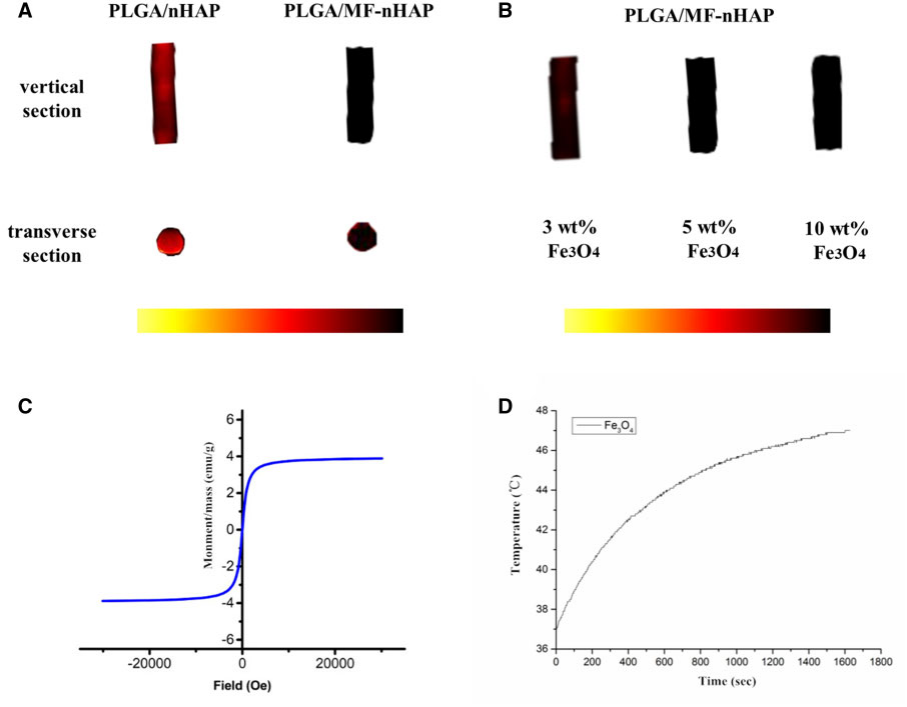
Magnetic and thermal properties. NMR results of PLGA/nHAP and PLGA/MF-nHAP scaffold (A). Characterization of different Fe_3_O_4_ contents of PLGA/MF-nHAP scaffold (B). Magnetic hysteresis loop of 5 wt% Fe_3_O_4_ scaffolds (C). Heating conditions of Fe_3_O_4_ particle (D).

T2-weighted imaging displayed that the signal of PLGA/MF-nHAP was significantly stronger than that of PLGA/nHAP (Fig. 2A). The content of Fe_3_O_4_ in PLGA/MF-nHAP scaffolds was increased from 3 wt% to 5 wt%. The signal was significantly enhanced, and when it was further increased to 10 wt%, there was no significant change in the signal. Therefore, the 5 wt% Fe_3_O_4_ scaffolds were selected for *in vivo* and *in vitro* studies (Fig. 2B). The result of magnetic test showed that magnetic saturation of 5 wt% Fe_3_O_4_ scaffolds was 3.89 emu/g (Fig. 2C). Temperature of Fe_3_O_4_ particle increased with the reaction time and reached ultimate stability in 47°C under the condition of 500 kHz frequency and 3 mT magnetic field intensity (Fig. 2D).

#### Mechanical property

**Figure 3:**
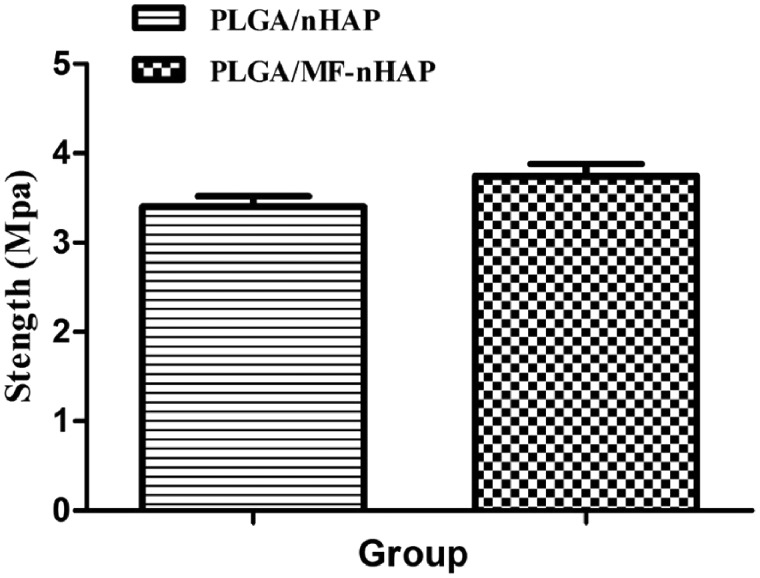
Mechanical properties of PLGA/nHAP and PLGA/MF-nHAP scaffolds.

The mechanical properties of the scaffolds were evaluated by compressive tests. The compressive strength of the PLGA/MF-nHAP slight higher than those of the PLGA/nHAP (3.40 ± 0.11 MPa versus 3.74 ± 0.14 MPa), but without statistically significant (Fig. 3).

### Biocompatibility of magnetite nanoparticles and composite scaffolds *in vitro*

#### Inactivation of tumor cells

**Figure 4:**
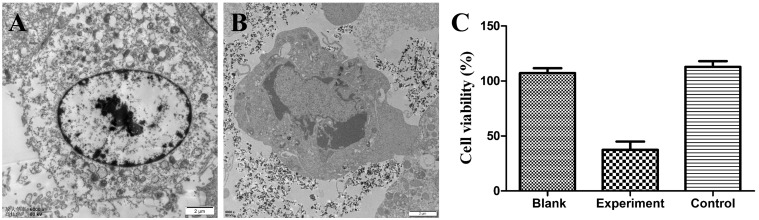
The TEM image of MG-63 cells incubated with magnetite nanoparticles (A) and after exposed to magnetic field for 30 min (B). CCK-8 assay of MG-63 cells viability with exposed to magnetic field for 30 min or not. Cells cultured with or without magnetite nanoparticles as blank and control, cells cultured with magnetite nanoparticles exposed to magnetic field for 30 min as experiment.

MG-63 cells (1 × 105) co-culture with nanoparticles were observed cell survival via TEM and measured cell viability via the CCK-8 assay. Without thermal treatment, the cell endocytosed nanoparticles without significant morphological change (Fig. 4A). After thermal treatment, cell morphological obvious changed, including chromatin condensed in margin and interruption of nuclear membrane continuity (Fig. 4B). CCK-8 assay displayed that 78% cells died after hyperthermia (Fig. 4C).

#### Cell adhesion and proliferation

**Figure 5:**
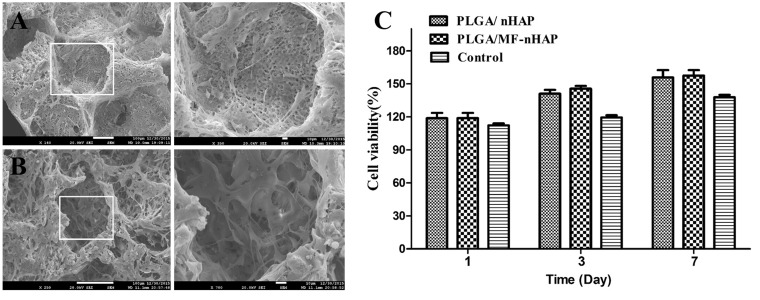
SEM images of MC3T3-E1 cells adhered to (A) PLGA/nHAP and (B) PLGA/MF-nHAP scaffolds after being cultured on them for 24 h. CCK-8 assay of MC3T3-E1 cells incubated on scaffolds for different periods (C). Cells cultured without scaffolds were calculated as control.

MC3T3-E1 cells seeded on the scaffolds were observed cell adhesion via SEM and measured cell viability via the CCK-8 assay. As shown in Fig. 5A and B, MC3T3-E1 cells adhered well to the both scaffolds without significant morphological change. Furthermore, cell proliferations on PLGA/MF-nHAP and PLGA/nHAP were a little more than those on the blank group according to the results of CCK-8 assay, but there were no statistically significant differences (Fig. 5C).

### Bone repair of scaffolds *in vivo*

#### X-ray examination of repaired bone defect

**Figure 6:**
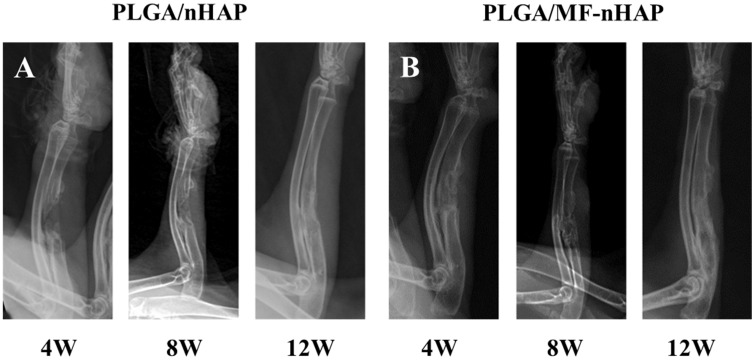
X-ray examination of (A) PLGA/nHAP and (B) PLGA/MF-nHAP scaffolds implanted in ulnas defect at different periods.

The bone repair effects of different scaffolds were observed by X-ray examination. As shown in Fig. 6A and B. Callus formation of bone defect occurred 4 weeks after surgery, the density is lower than normal surrounding bone tissue. By 8 weeks, the outline of the medial cortical appeared and the density of callus became blurry and uneven. To 12 weeks, the density of callus tends to be uniform and the bone defect is basically healed. There were no differences in the bone repair performances of PLGA/MF-nHAP and PLGA/nHAP at different time intervals.

#### Micro-CT scan of repaired bone defect

**Figure 7:**
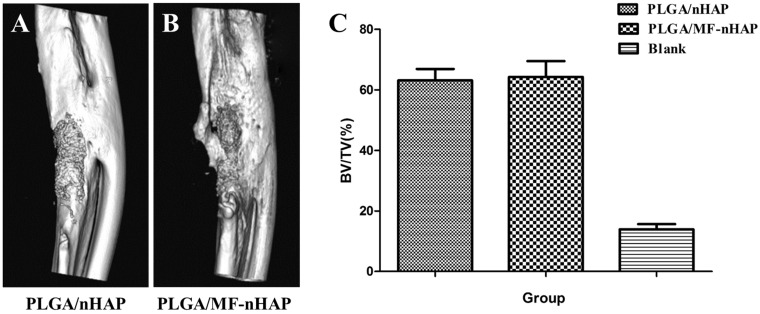
3D reconstruction image of (A) PLGA/nHAP and (B) PLGA/MF-nHAP scaffolds implanted in ulnas defect at 12 weeks. BV/TV of defect regions was calculated between three groups (C).

Micro-CT reconstructions of the ulnas defects in rabbits implanted with PLGA/MF-nHAP and PLGA/nHAP at 12 weeks are shown in Fig. 7A and B. On the whole, PLGA/MF-nHAP and PLGA/nHAP showed prominent new bone formation compared with blank. BV/TV of defect regions was displayed in Fig. 7C. At 12 weeks after implantation, the BV/TV of PLGA/nHAP (63.12 ± 7.56) and PLGA/MF-nHAP (64.26 ± 10.44) was significantly higher than that of blank (13.88 ± 3.58) and the difference was statistically significant.

#### Histology analysis

**Figure 8:**
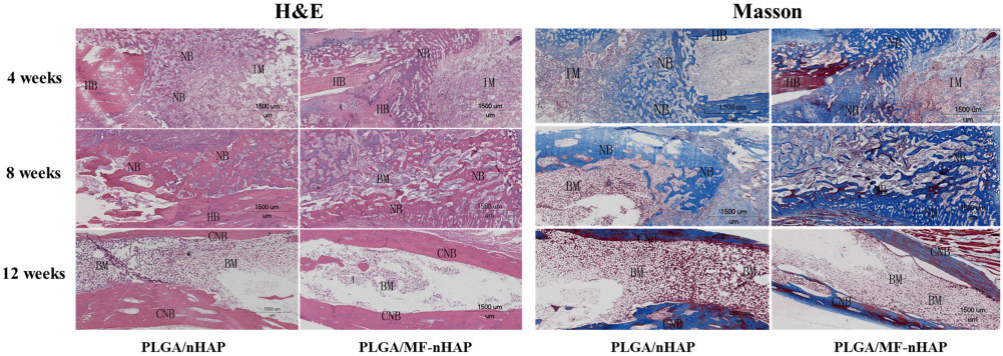
Histological examination of the ulnas defects repaired by PLGA/nHAP and PLGA/MF-nHAP at 4, 8 and 12 weeks after implantation. HB, host bone; NB, new bone; IM, implant material; BM, bone marrow; CNB, cortical bone.

To further obtain an insight into the bone repair effects of various scaffolds, implanted materials were stained with H&E and masson. As indicated in Fig. 8, H&E staining displayed that both PLGA/MF-nHAP and PLGA/nHAP scaffolds were partially degraded in the 4 weeks after surgery and the combined part had new bone formation. After 8 weeks, new bone formation increased while the implanted materials decreased, and woven bone appeared in the defect area. To 12 weeks, the partial remodeling of the new bone into cortical bone and medullary cavity recanalization. The same result exhibited with masson staining, new bone formation increased while the implanted materials decreased during bone repair. Partial blue trabeculae of new bone can be seen in the defect area 4 weeks after surgery and became dark blue and weaving shaped at 8 weeks. Arrive at 12 weeks, the new bone is joined in sheets, showing varying degrees of redness, indicating a transition from new to mature bone.

## Discussion

Bone defect is a common problem encountered in the treatment of musculoskeletal tumor surgery. Not only to inhibit tumor growth and recurrence, but also to repair the defect and preserve the limb function. Hence, it is necessary to find an ideal functional biomaterial that can repair bone defects and inhibit tumor cell growth.

In this study, we prepared Fe_3_O_4_ MNPs and combined it with PLGA-COOH/HAP scaffolds. The diameter of MNPs prepared by us was about 5 nm, far smaller than the critical particle size with the characteristics of superparamagnetic [25]. In addition, the magnetic hyperthermia temperature of Fe_3_O_4_ particles could stabilize at 47°C, which was above the tolerance temperature of tumor cells and within the tolerance range of normal cells [26]. Cell experiment confirmed it can effective kill tumor cells, which provides a reference for the repair of bone defect during the treatment of bone tumor.

In the early stage, we proved that the PLGA/nHAP scaffold had excellent bone regeneration ability [23]. When combined with Fe_3_O_4_ particles, the particles are uniformly distributed and possess NMR imaging. In addition, the addition of Fe_3_O_4_ particles to the scaffold does not affect its microstructure, and it also had good porous structure and excellent porosity, which simulated bone morphology and promoted molecular interactions with cells [27]. The mechanical properties of both scaffolds meet the needs of the implant materials, and the compressive strength of PLGA/MF-nHAP scaffold was little higher than PLGA/nHAP scaffold, which may be that the addition of particles enhances the scaffold microstructure.

It has been confirmed that PLGA/nHAP composite scaffolds have good biocompatibility. Introduction of Fe_3_O_4_ particles to the scaffolds did not show the cytotoxicity. On contrary, CCK-8 assay displayed that the number of viable cells cultured on the PLGA/MF-nHAP scaffold was slightly higher than that on the PLGA/nHAP scaffold. That could be attributed to Fe_3_O_4_ particles, which were found in some studies that it could promote hBMCs adhesion and proliferation and increased osteoblast functions with or without a magnetic field [28, 29]. In addition, Quan *et al*. [30] prepared a new multifunctional that HAP is synthesized by spontaneous assembly of alendronate and Fe_3_O_4_ onto HAP nanocrystals, for osteoporotic bone regeneration, it can inhibit osteoclastic activity and promote osteoblast proliferation and differentiation. This indicated that Fe_3_O_4_ alone or in combination with other materials was beneficial to enhance cell activity.

The capability of bone regeneration of PLGA/MF-nHAP scaffold in ulnas defect of rabbits also have been evaluated. X-ray dynamic monitoring of bone growth of bone defects and micro-CT reconstructions of the ulnas directly confirmed that PLGA/MF-nHAP scaffold had good osteogenic performance. H&E and masson dyeing were further affirmed these newly formed bone tissues. At 12 weeks, part of the new bone was remodeled to form cortex and part of the medullary cavity was recanalized. That means adding the Fe_3_O_4_ particles, the scaffold did not affect bone regeneration capability compared with PLGA/nHAP scaffold. Zhao *et al*. [31] confirmed Fe_3_O_4_ added in chitosan/collagen/nHAP scaffold possessed a better tissue compatibility and higher bone regeneration ability in rat skull defects.

However, there was also a limitation to our study. We did not evaluate the antitumor ability of the composite scaffolds *in vitro* and *in vivo*, because it was difficult to fabricate specific devices to provide the required magnetic field. The data of antitumor assay of MNPs obtained *in vitro* could be used as a reference, further methods needed more research on the effects of antitumor of composite scaffolds.

## Conclusions

In summary, we successfully prepared Fe_3_O_4_ nanoparticles and PLGA/MF-nHAP scaffold. In vitro experiments, magnetic particles have been showed good thermal effect and antitumor ability. When combining it to the PLGA/nHAP, it also possessed good biocompatibility and bone regeneration capacity. PLGA/MF-nHAP scaffold can not only enhance cell adhesion and proliferation, but also effectively promote the repair of radial defect. Our research has been verified that PLGA/MF-nHAP scaffold has a good potential in bone repair, and it is necessary to further study the effect of bone repair on bone defect after tumor resection.
